# Fabrication and Characterization of Ferroelectric Capacitors with a Symmetric Hybrid TiN/W/HZO/W/TiN Electrode Structure

**DOI:** 10.3390/ma18153547

**Published:** 2025-07-29

**Authors:** Ha-Jung Kim, Jae-Hyuk Choi, Seong-Eui Lee, So-Won Kim, Hee-Chul Lee

**Affiliations:** Department of Advanced Materials Engineering, Tech University of Korea, Siheung 15073, Republic of Korea; 2023811075@tukorea.ac.kr (H.-J.K.); gur7226@tukorea.ac.kr (J.-H.C.); selee@tukorea.ac.kr (S.-E.L.)

**Keywords:** HZO ferroelectric capacitor, TiN electrode, W electrode, rapid thermal annealing, high remnant polarization, leakage current

## Abstract

In this study, Hf_0.5_Zr_0.5_O_2_ (HZO) thin-films were deposited using a Co-plasma atomic layer deposition (CPALD) process that combined both remote plasma and direct plasma, for the development of ferroelectric memory devices. Ferroelectric capacitors with a symmetric hybrid TiN/W/HZO/W/TiN electrode structure, incorporating W electrodes as insertion layers, were fabricated. Rapid thermal annealing (RTA) was subsequently employed to control the crystalline phase of the films. The electrical and structural properties of the capacitors were analyzed based on the RTA temperature, and the presence, thickness, and position of the W insertion electrode layer. Consequently, the capacitor with 5 nm-thick W electrode layers inserted on both the top and bottom sides and annealed at 700 °C exhibited the highest remnant polarization (2P_r_ = 61.0 μC/cm^2^). Moreover, the symmetric hybrid electrode capacitors annealed at 500–600 °C also exhibited high 2P_r_ values of approximately 50.4 μC/cm^2^, with a leakage current density of approximately 4 × 10^−5^ A/cm^2^ under an electric field of 2.5 MV/cm. The findings of this study are expected to contribute to the development of electrode structures for improved performance of HZO-based ferroelectric memory devices.

## 1. Introduction

Recently, the development of ferroelectric materials for applications in next-generation non-volatile memories and low-power transistors has gained considerable momentum. In particular, HfO_2_-based thin-films have attracted attention owing to their excellent compatibility with CMOS processes and their ability to exhibit ferroelectricity even at thicknesses of just a few nanometers [1,2,3,4]. Although the monoclinic (m-phase) structure—which is centrosymmetric and non-ferroelectric—is the thermodynamically stable phase of HfO_2_-based films at room temperature, phase transitions to the non-centrosymmetric orthorhombic (o-phase) with ferroelectric properties can occur in ultrathin films (~10 nm) owing to the relatively low kinetic barrier. Accordingly, extensive research has been conducted to promote o-phase formation through the optimization of the dopant type, annealing conditions, and electrode materials [5,6,7,8].

In a previous study, our research team deposited Hf_0.5_Zr_0.5_O_2_ (HZO) films using a Co-plasma atomic layer deposition (CPALD) process that simultaneously used both direct plasma (DP) and remote plasma (RP). We then analyzed the electrical properties of devices fabricated in a metal–ferroelectric–metal (MFM) structure based on these films [9]. The results showed that CPALD-deposited films exhibited a more efficient phase-transition to the ferroelectric o-phase during post-deposition annealing compared to films deposited via DPALD. Moreover, these films demonstrated higher remnant polarization and superior fatigue endurance characteristics. These improvements could be attributed to the high-energy radicals supplied during CPALD, which reduced the carbon residue while mitigating plasma-induced damage owing to the relatively mild plasma conditions.

One of the key factors in suppressing the m-phase and inducing a transition to the o-phase in HZO thin-films is the tensile stress and associated strain ($\epsilon_{T}$) imposed on the film by the mismatch in the thermal expansion coefficients (TECs) between the capping electrode material and the film during the cooling process after annealing. This relationship can be expressed as follows [10,11,12]:

(1) $$\epsilon_{T}\left(T_{A}\right)=\int_{RT}^{T_{A}}\left(\alpha_{film}-\alpha_{cap}\right)dT$$

where *α_film_* denotes the TEC of the film, *α_cap_* denotes the TEC of the electrode, and *T_A_* and *RT* denote the annealing temperature and room temperature, respectively.

To date, much research has focused on using single conductive nitride electrodes such as TiN—commonly used in semiconductor devices—to induce o-phase formation. However, TiN(α~9.4 × 10^−6^ K^−1^) has a relatively small TEC mismatch with HZO(α~10 × 10^−6^ K^−1^), limiting the stress-induced effect. Moreover, during annealing TiN can be prone to oxidation in the HZO deposition environment, leading to the formation of an interfacial TiO_x_ layer which degrades the polarization stability and overall device performance [13,14,15,16,17].

Therefore, in this study the term hybrid electrode refers to a bilayer metal structure consisting of W and TiN, in which a low TEC W layer (α = 4.4 × 10^−6^ K^−1^) is inserted into the conventional MFM structure based on HZO films deposited by CPALD. A symmetric hybrid electrode configuration, TiN/W/HZO/W/TiN, was realized by applying the same W/TiN bilayer structure to both the top and bottom electrodes. This structural improvement aims to enhance electrical performance and facilitate future memory device applications. Initially, we analyzed the crystallographic phase and electrical properties of the films with respect to the annealing temperature in the presence of W insertion layers. Subsequently, we investigated how the thickness and arrangement of the W hybrid electrode influenced the structural and electrical characteristics of HZO-based ferroelectric capacitors.

## 2. Experimental Methods

**Figure 1:**
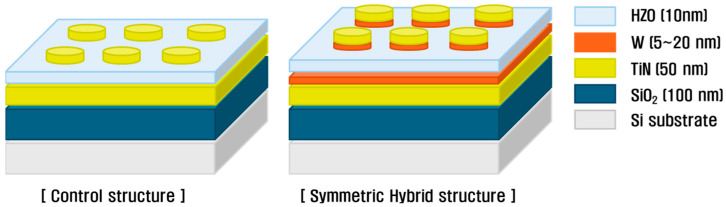
Schematic of an MFM capacitor structure with a W insertion electrode layer.

To fabricate the MFM capacitor structure, a substrate consisting of a SiO_2_ (100 nm)/p-type Si wafer with a 50 nm-thick TiN bottom electrode was used. HZO thin-films were deposited on the substrate using a plasma-enhanced atomic layer deposition (PEALD, iOV-dx2, iSAC Research, Daejeon, Republic of Korea) system, applying the CPALD technique, which simultaneously used DP and RP discharges. For the deposition process, tetrakis(ethylmethylamino) hafnium (TEMAH, iChems, Hwaseong, Republic of Korea) and tetrakis(ethylmethylamino) zirconium (TEMAZ, iChems, Hwaseong, Republic of Korea) were used as the Hf and Zr precursors, respectively. Alternating ALD cycles of HfO_2_ and ZrO_2_ were applied to achieve a 1:1 ratio, forming a 10 nm-thick HZO thin-film. The deposition temperature was maintained at 240 °C, and O_2_ was used as the reactant gas. During the reactant gas injection process, the DP was activated by applying 100 W RF power for 2 s through a plasma generator inside the chamber, whereas the RP was simultaneously activated using an external remote plasma system (RPS, En2ra-RPS, EN2CORE Technology, Daejeon, Republic of Korea), applying 2.6 kW power for 12 s to generate a high-density plasma. The top electrode was fabricated via a lift-off process to form 200 μm-diameter, 50 nm-thick, dot-shaped TiN single electrodes or W/TiN hybrid electrodes, using RF magnetron sputtering. When introducing a W insertion layer above the HZO ferroelectric film, the W layer was deposited first, followed by in situ TiN deposition. By varying the W insertion layer thickness from 0 to 20 nm, a symmetric hybrid TiN/W/HZO/W/TiN ferroelectric capacitor structure was fabricated, as illustrated in Figure 1. Finally, rapid thermal annealing (RTA) was performed to crystallize the film using a nitrogen atmosphere at temperatures ranging from 500 to 700 °C for 30 s.

The crystallinity of the HZO thin-films deposited by CPALD was analyzed using X-ray diffraction (XRD, SmartLab, Rigaku, Tokyo, Japan), and the chemical bonding states were examined by X-ray photoelectron spectroscopy (XPS, AXIS-NOVA, Manchester, UK). The cross-sectional morphology and crystallization of the films were observed using field emission transmission electron microscopy (FE-TEM, JEM-2100F, JEOL, Tokyo, Japan). For electrical characterization of the fabricated MFM devices, a semiconductor parameter analyzer (4200-SCS, Keithley, Cleveland, OH, USA) connected to a microprobe station (APX-6B, WIT Co., Suwon, Republic of Korea) was used to measure the P–E (polarization–electric field) hysteresis curves and I–V (current–voltage) curves. Measurements were conducted using a ±3 V triangular pulse at 1 kHz frequency, and measurements were taken before and after 10^4^ cycles to account for the wake-up effect of the films [18,19].

## 3. Results and Discussion

**Figure 2:**
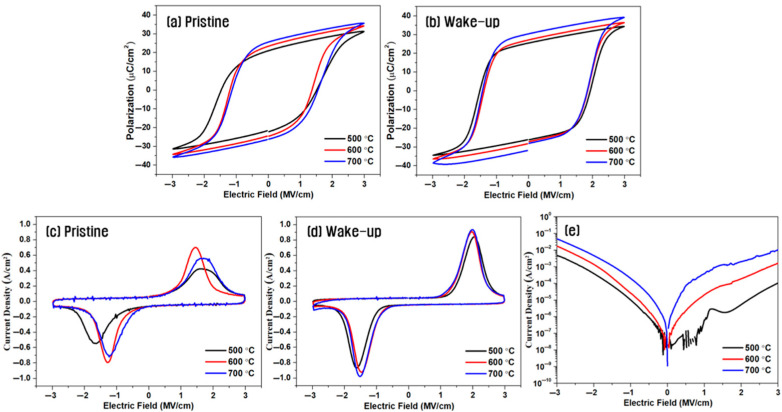
P–E hysteresis loops (**a**,**b**), switching current (**c**,**d**), and leakage current (**e**) of capacitors with a symmetric hybrid TiN/W/HZO/W/TiN structure with a 10 nm-thick W insertion layer at various annealing temperatures; (**a**,**c**) in the pristine state and (**b**,**d**) in the wake-up state.

Figure 2 presents the electrical characteristics of capacitors with a symmetric hybrid TiN/W/HZO/W/TiN electrode structure, where 10 nm-thick W insertion electrodes were incorporated at both the top and bottom interfaces of the HZO layer. The devices were subjected to post-deposition annealing at various temperatures. Figure 2a,b show the P–E hysteresis loops in the pristine state and after 10^4^ wake-up cycles, respectively. Devices with the W insertion layers exhibited excellent ferroelectric behavior in the pristine state regardless of the annealing temperature, with 2P_r_ values ranging from 43 to 52 μC/cm^2^. Upon wake-up cycling, the remnant polarization and coercive field increased, producing ideal square-shaped hysteresis loops. Notably, the sample annealed at 700 °C exhibited the highest remnant polarization with a 2P_r_ of 60.5 μC/cm^2^.

Figure 2c,d show the corresponding switching current density curves. Symmetrical double switching peaks were already evident before wake-up, and well-defined switching current peaks appeared near the coercive field (*E_c_*) after wake-up cycling, indicating enhanced ferroelectric performance.

Figure 2e shows the leakage current characteristics of the same samples as a function of annealing temperature. The leakage current slightly increased with higher annealing temperatures, likely owing to the diffusion of W metal species and increased oxygen vacancy concentration, promoting charge transport along the grain boundaries [20,21].

**Figure 3:**
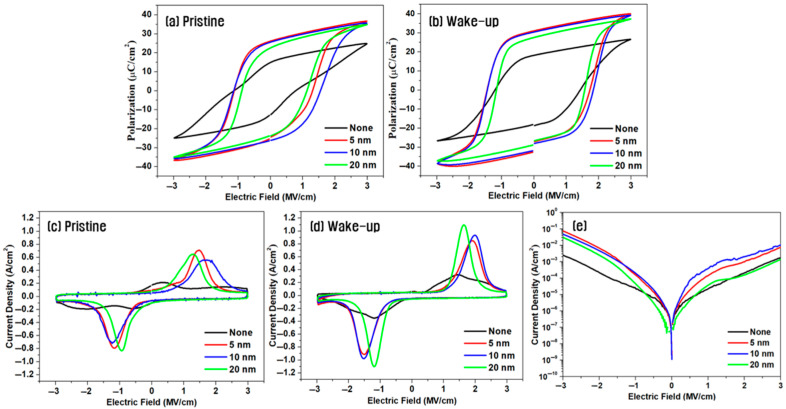
P–E loops (**a**,**b**), switching current (**c**,**d**), and leakage current (**e**) of capacitors with a symmetric electrode structure annealed at 700 °C, as a function of the W insertion layer thickness; (**a**,**c**) in the pristine state and (**b**,**d**) in the wake-up state.

Figure 3 shows the effect of varying the thickness of the W insertion layer on the electrical properties of capacitors with the symmetric electrode structure annealed at 700 °C. As shown in Figure 3a,b, the device without W insertion exhibited a 2P_r_ of 27.8 μC/cm^2^ in the pristine state, which increased to 36.7 μC/cm^2^ after 10^4^ wake-up cycles. By contrast, the hybrid electrode device with a 5 nm-thick W layer exhibited a high 2P_r_ of 51.2 μC/cm^2^ even before wake-up, which further increased to 61.0 μC/cm^2^ after wake-up—the highest value observed in this study. With a 10 nm-thick W insertion layer the 2P_r_ remained high at 60.5 μC/cm^2^ after wake-up, whereas with a 20 nm-thick layer the value decreased slightly to 55.4 μC/cm^2^. This decline could be attributed to the emergence of bulk-like behavior rather than thin-film characteristics as the W layer became too thick. The increase in W layer thickness may induce subtle changes in crystallographic orientation and the corresponding surface energy, which can contribute to a reduced formation of the o-phase and a degradation of ferroelectric properties [22,23].

Figure 3c,d show the switching current measurements. In the absence of the W insertion layer, the switching current in the pristine state exhibited weak double peaks, whereas devices with W layers exhibited clearly defined switching peaks. After wake-up cycling, all devices exhibited enhanced ferroelectricity, and the switching current peak areas were largest in devices with 5–10 nm-thick W layers, consistent with the observed remnant polarization values.

As shown in Figure 3e, the leakage current increased by up to one order of magnitude in devices with W insertion layers. This increase is believed to result from the diffusion of W atoms into the HZO film during annealing, in which the presence of metallic ions in the oxide matrix reduces the insulation quality and thus increases the leakage current [24,25,26].

**Figure 4:**
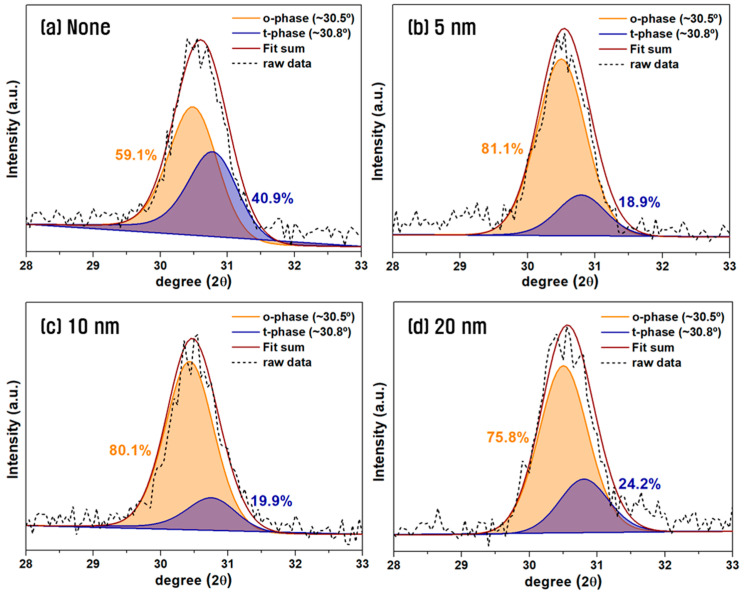
XRD patterns of HZO films with different W insertion thicknesses after 700 °C RTA in a symmetric electrode structure.

Figure 4 shows the XRD analysis results of HZO thin-films in the symmetric electrode structure from Figure 3 as a function of the W insertion layer thickness. XRD analysis was conducted over the 2θ range of 28–33°, and Gaussian deconvolution was applied around the o-phase (~30.5°) and t-phase (~30.8°) to fit the diffraction patterns. The relative ratio of o-/t-phase was determined by integrating the area under each fitted peak [27,28,29]. In the TiN/HZO/TiN structure without a W insertion layer, the o-phase fraction decreased from 62.6% at 600 °C to 59.1% at 700 °C, consistent with the general trend that higher annealing temperatures favor the thermodynamically stable t-phase and suppress o-phase formation [30,31,32]. By contrast, when the W insertion layer was used the o-phase fraction increased with increasing annealing temperature, regardless of the W layer thickness. This can be attributed to the greater TEC mismatch between the HZO and W (α = 4.4 × 10^−6^ K^−1^) compared to TiN, resulting in greater tensile stress that helps stabilize the o-phase [33,34]. Based on a simple calculation using Equation (1), the tensile strain induced by the TEC mismatch at the W/HZO and TiN/HZO interfaces under a 700 °C annealing condition is estimated to be approximately 0.38% and 0.04%, respectively. This difference may be significant in promoting the phase transformation into the metastable ferroelectric phase [35]. Notably, the o-phase fraction peaked at 81.1% when the W insertion layer was 5 nm-thick and remained high at 80.1% with a 10 nm-thick W layer. However, it decreased to 75% when a 20 nm-thick W insertion was used. This trend was consistent with the 2P_r_ values observed in the electrical measurements.

**Figure 5:**
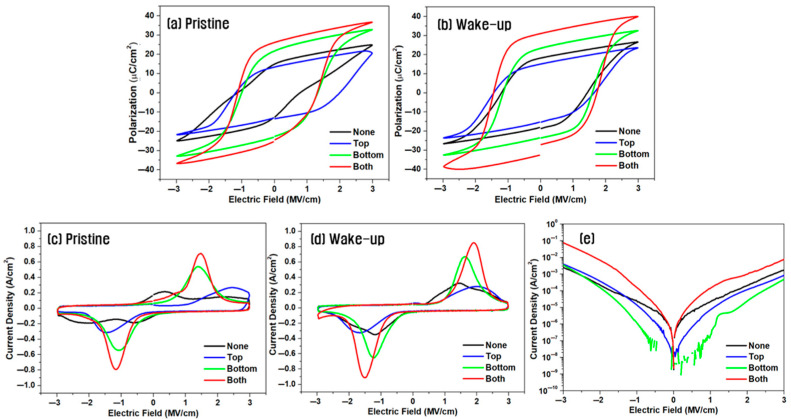
P–E loops (**a**,**b**), switching current (**c**,**d**), and leakage current (**e**) of capacitors with various electrode configurations using 5 nm-thick W insertion layer; (**a**,**c**) in the pristine state and (**b**,**d**) in the wake-up state.

Figure 5 presents the electrical characterization of capacitors with various electrode configurations, each using a 5 nm-thick W insertion layer. As shown in Figure 5a, comparing the pristine states, devices without a W layer, or with a W layer only at the top electrode, exhibited low 2P_r_ values and weak polarization curves. By contrast, configurations with the W layer at the bottom electrode, or at both the top and bottom, exhibited strong ferroelectric behavior with high 2P_r_ values.

As shown in Figure 5b, the wake-up state measurements show well-formed ferroelectric hysteresis curves in all configurations. The dual W insertion structure (top and bottom) produced the highest 2Pr value of 61 μC/cm^2^. The asymmetric configuration with W only at the bottom yielded the second highest value of 47 μC/cm^2^, whereas placing W only at the top resulted in a lower 2P_r_ of 30.5 μC/cm^2^.

The switching current results shown in Figure 5c,d reinforce this trend. Devices without W, or with top-only W layers, exhibited weak, asymmetric switching current peaks in the pristine state. By contrast, devices with W at the bottom, or at both interfaces, exhibited distinct polarization current peaks. After wake-up, these devices exhibited larger peak areas and more symmetric switching currents, consistent with enhanced ferroelectric performance. Notably, in the device incorporating W electrodes on both sides, the positions of the two switching current peaks are shifted toward the positive and negative directions, respectively. The shift reflects an increase in the coercive field, implying that a higher electric field is required to induce polarization reversal. We know that W is a metal with excellent oxidation resistance and low reactivity at the HZO interface, effectively suppressing the formation of undesirable interfacial oxide layers [36,37,38]. In typical TiN/HZO structures, oxygen supplied during HZO deposition can react with the bottom TiN electrode to form TiO_x_ interfacial layers, which can be expected to be thicker than those at the top interface. These interfacial oxides can alter the electron affinity and the intrinsic band structure at the interface. In particular, the conduction band minimum at the bottom interface can rise more than that at the top, increasing the conduction band offset (CBO). If this CBO becomes too large, it can concentrate the electric field at the interface, leading to the breakdown and degradation of electrical properties [39,40,41]. Consequently, inserting W at the bottom electrode can be expected to more effectively suppress interfacial oxide growth and stabilize the CBO, contributing more to device performance than top-only W insertion.

Figure 5e shows the leakage current results. The device with W only at the bottom electrode exhibited a lower leakage current compared to those with no W or top-only W insertion. The highest leakage current was evident in the dual-insertion device, likely owing to the excessive diffusion of W during high-temperature annealing.

**Figure 6:**
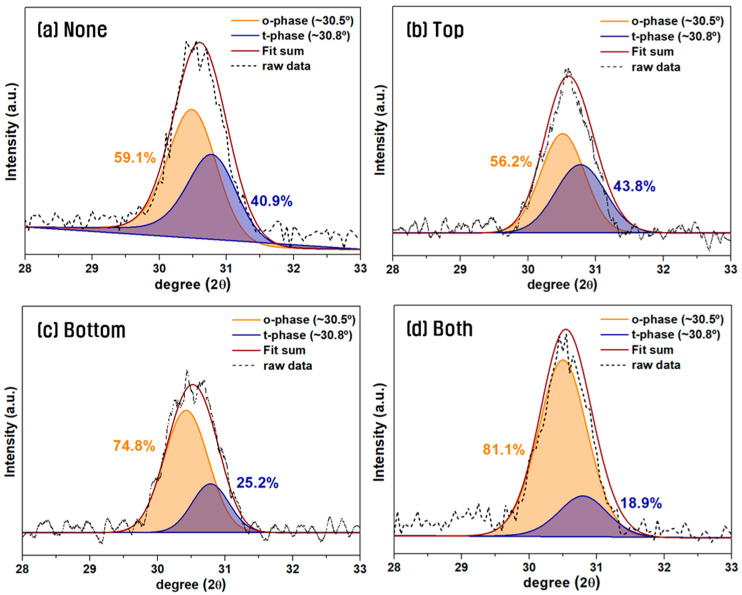
XRD patterns of HZO films with four different electrode configurations using 5 nm-thick W insertion layer.

Figure 6 shows the XRD analysis results of HZO films with the different electrode configurations described in Figure 5. When W was inserted only at the top electrode, the o-phase fraction was even lower than that of the device without any W insertion. By contrast, inserting W at the bottom electrode, or at both electrodes, increased the o-phase fraction to 74.8% and 81.1%, respectively. As in the previous results, a strong correlation was evident between the o-phase fraction and the 2Pr values measured in Figure 5, confirming a proportional relationship.

**Figure 7:**
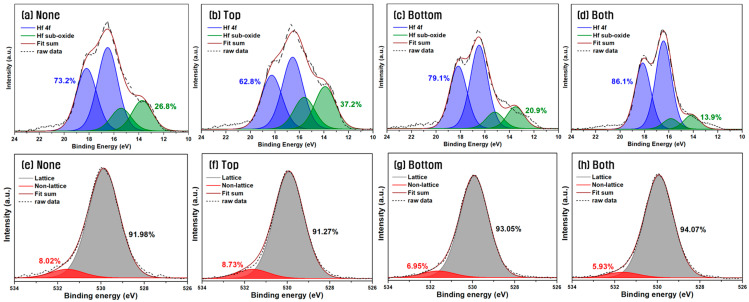
Comparison of narrow-scan XPS spectra of (**a**–**d**) Hf 4f and (**e**–**h**) O 1s for different electrode configurations.

To analyze the cause of the crystallinity and electrical property variations depending on the W insertion position in the MFM device structure, XPS analysis was conducted. The XPS measurements were performed using a depth profiling method, in which the samples were etched 20 times after full device fabrication until reaching the bottom TiN electrode. Spectral analysis was based on the region within the HZO film where the relative concentration of Hf and Zr was the highest. Figure 7 shows the XPS narrow-scan spectra of Hf 4f and O 1s for the HZO films corresponding to the devices shown in Figure 5 and Figure 6, with different W insertion layer positions. In Figure 7a–d, the Hf 4f peaks were deconvoluted using a Gaussian fitting method with the intensity of 4f_7/2_:4f_5/2_ fixed at 4:3, a spin–orbit splitting of 1.66 eV, and identical FWHM constraints [42]. The proportion of non-stoichiometric HfO_2−x_—indicative of lattice defects in HfO_2_—was 26.8% in the sample without a W insertion layer, whereas with W inserted at the bottom, or at both interfaces, the ratio decreased to 20.9% and 13.9%, respectively.

The O 1s peak narrow-scan analysis shown in Figure 7e–h was used to examine the proportion of non-lattice oxygen, which corresponds to oxygen vacancies in the HZO film. Although the non-lattice component may include various oxygen-related species such as O-H and C-O, in addition to oxygen vacancies, no distinct peaks corresponding to C–O (286.5 eV) or O–C=O (288.5 eV) were observed in the C 1s spectrum acquired at the same depth, thereby excluding the possibility of contamination from C–O-related species. Compared to the 8.02% non-lattice oxygen in the film without a W insertion layer, the ratios dropped to 6.95% and 5.93% when W was inserted at the bottom and both electrodes, respectively. These results indicate that placing the W insertion layer at the bottom, or at both interfaces, reduces the presence of non-stoichiometric Hf species and non-lattice oxygen components. This trend is consistent with the previously observed increase in 2P_r_ and the change in the o-/t-phase ratio. Therefore, the reduction in defect states is believed to have played a crucial role in the structural stability of the HZO film and the enhancement in the electrical performance of HZO-based capacitors [43,44,45,46].

In particular, when W was inserted only at the top electrode, the defect ratio increased, which supports the earlier interpretation that inserting W at the bottom electrode effectively suppresses interfacial oxide formation and stabilized the CBO, thereby having a more favorable impact on device performance. These findings suggest that applying the W insertion layer to the bottom electrode, rather than the top, may more significantly contribute to the structural and electrical enhancement of the device.

**Figure 8:**
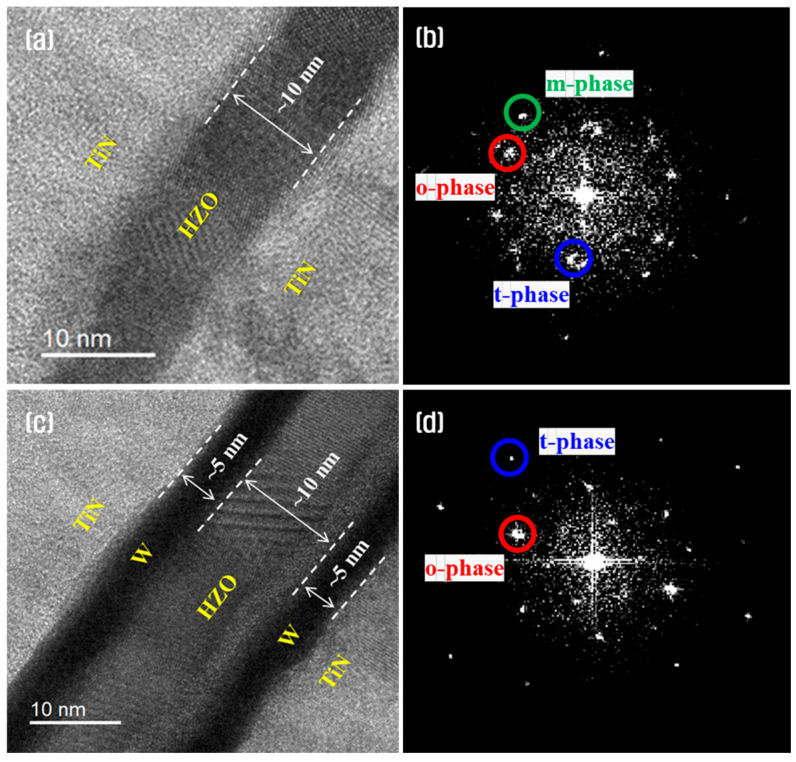
Cross-sectional, high-resolution TEM images (**a**,**c**) and corresponding FFT patterns (**b**,**d**) of the HZO thin-films with and without a W insertion layer.

Figure 8 shows the high-resolution, cross-sectional TEM images and corresponding fast Fourier transform (FFT) patterns of the HZO thin-films with and without a W insertion layer. In both films, well-defined interfaces and sufficient crystallization could be observed; however, the interface between the HZO and TiN was smoother and more distinct when a W insertion layer was used. Regardless of the presence of W, the HZO films had similar thicknesses under identical ALD cycle conditions.

Figure 8b,d reveal that in the absence of a W insertion layer diffraction patterns corresponding to the m-phase (with a d-spacing of 3.16 Å) were evident in addition to the o-phase and t-phase peaks. By contrast, in the presence of a W insertion layer only the o-phase (2.65 Å) and t-phase (2.92 Å) patterns could be identified. This suggests that the W layer effectively suppressed interfacial reactions between HZO and TiN, promoted o-phase transition, and successfully inhibited undesirable m-phase formation [47,48].

**Figure 9:**
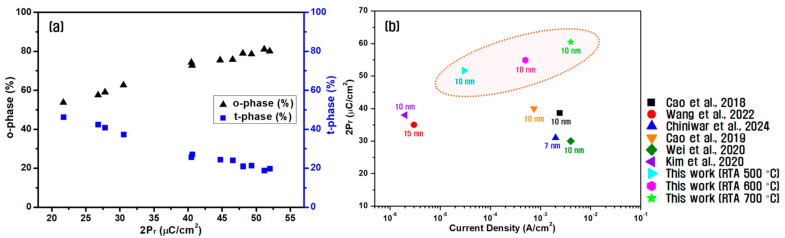
(**a**) Variation in the o- and t-phase fractions of the fabricated HZO films as a function of 2Pr and (**b**) comparison of the electrical performance of the developed hybrid TiN/W/HZO/W/TiN capacitors with previously reported devices without a W insertion layer [10,37,46,52,53,54].

Figure 9a shows the variation in the o-phase and t-phase fractions of all fabricated HZO capacitors as a function of their measured 2P_r_ values. As 2P_r_ increased, the o-phase fraction increased linearly, whereas the t-phase fraction decreased correspondingly. This confirms that an increased proportion of the metastable o-phase is the key to strong ferroelectric performance in HZO films [49,50,51].

Figure 9b compares the electrical performance—specifically the polarization and leakage current—of the developed symmetric hybrid TiN/W/HZO/W/TiN capacitors with those of previously reported TiN/HZO/TiN or W/HZO/W capacitors without W insertion layers. Most reference devices were annealed at 500 °C, conditions under which the capacitor fabricated in this study exhibited the highest 2P_r_ value. Here, 2P_r_ increased with higher annealing temperatures. The leakage current values were also lower or comparable to other devices with similar HZO thickness, demonstrating that the use of a W insertion layer could simultaneously improve the polarization performance and suppress the leakage current.

## 4. Conclusions

In this study, HZO thin-films were deposited using a CPALD process that simultaneously used DP and RP. The influence of the thickness and position of W insertion layers in a symmetric hybrid electrode configuration on the electrical performance of CPALD HZO ferroelectric capacitors was investigated. Under 700 °C annealing conditions, the device with 5 nm-thick W insertion layers applied at both the top and bottom electrodes exhibited excellent ferroelectric properties, even before wake-up cycling. After wake-up the capacitor exhibited a remarkably high remnant polarization of 2P_r_ = 61.0 μC/cm^2^. The position of the W insertion layer clearly affected the device characteristics—for example, inserting W at the bottom electrode resulted in higher 2P_r_ values and a considerably reduced leakage current compared to top-only insertion. This improvement could be attributed to the large TEC mismatch between W and HZO, which generated tensile stress, and to the suppression of interfacial oxide layer formation caused by reactions between the bottom TiN electrode and oxygen during the deposition process. Overall, the W insertion layer contributed to improved electrical performance by stabilizing the o-phase within the HZO film and reducing the formation of interfacial defects. The CPALD technique and the optimization of hybrid electrode configurations using W insertion layers have demonstrated promising potential for enhancing the performance of ferroelectric capacitors. Furthermore, for such devices to be viable candidates for next-generation memory applications, it is essential to achieve high cycling endurance and long retention times. To this end, we are currently preparing follow-up studies focused on addressing these critical reliability metrics.

## Data Availability

The original contributions presented in this study are included in the article. Further inquiries can be directed to the corresponding authors.

## Abbreviations

The following abbreviations are used in this manuscript:

ALD	Atomic layer deposition
CBO	Conduction band offset
CPALD	Co-plasma atomic layer deposition
DP	Direct plasma
FE-TEM	Field emission transmission electron microscopy
FFT	Fast Fourier transform
MFM	Metal–ferroelectric–metal
PEALD	Plasma-enhanced atomic layer deposition
RP	Remote plasma
RPS	Remote plasma system
RTA	Rapid thermal annealing
TEC	Thermal expansion coefficient
TEM	Transmission electron microscopy
XPS	X-ray photoelectron spectroscopy
XRD	X-ray diffraction
